# Biomechanical Characteristics of Scapular and Glenohumeral Movements during Pitching Motion in Injury-prone College Baseball Pitchers

**DOI:** 10.1298/ptr.E10254

**Published:** 2023-10-27

**Authors:** Koji MIYASHITA, Sentaro KOSHIDA, Taro KOYAMA, Kenichiro OTA, Yusuke TANI, Ryoji OKAMUNE

**Affiliations:** ^1^Department of Physical Therapy, College of Life and Health Sciences, Chubu University, Japan; ^2^Department of Judotherapy and Sports Medicine, Faculty of Health Sciences, Ryotokuji University, Japan; ^3^Matsushita Orthopedics, Japan; ^4^Watanabe Orthopedics and Rehabilitation Clinic, Japan; ^5^Advanced Reha Co. Ltd., Japan

**Keywords:** Scapula movement, Glenohumeral joint movement, Pitching motion, Injury-prone

## Abstract

Objectives: The coordination of glenohumeral (GH) and scapular movements is central to the injury prevention of baseball pitchers. However, there is no objective data establishing the direct relationship between pitching injuries and associated GH and scapular movements. Therefore, this study demonstrated the biomechanical differences in the scapular and GH movements during pitching between injury-prone pitchers and healthy college baseball pitchers.

Methods: A total of 30 collegiate baseball pitchers were classified into two groups according to their injury status: injury-prone group (n = 15) and control group (n = 15). We obtained pitching motion data using three-dimensional motion analysis technique.

Results: The horizontal abduction angles of the GH joint during cocking and acceleration phases were considerably greater in the injury-prone pitchers (19.0° at stride foot contact [SFC], −4.0° at maximum external rotation [MER], and −0.3° at ball release) than those in healthy controls (11.7° at SFC, −10.0° at MER, and −6.9° at ball release). Additionally, in the cocking phase, the amount of angular change in the scapular external rotation (ER) was significantly smaller in the injury-prone group than that in the control group (mean difference, −13.0).

Conclusion: These results suggest that the injury-prone pitchers have less internal rotation of the scapula and a more horizontal abduction of the GH joint during the cocking and acceleration phases. Therefore, sports medicine practitioners may need to pay considerable attention to the coordination of scapular and GH horizontal movements during pitching for prevention of shoulder injuries.

**T**he scapula plays a pivotal role in the kinetic chain, transferring energy derived from the trunk rotation to the pitching arm^1,2)^. Therefore, scapular dysfunction is believed to lead baseball pitchers to injuries, often occurring in the glenohumeral (GH) joint^3,4)^. However, despite evidence suggesting a link between the scapula dysfunction and shoulder pain, there is no consensus on a direct relationship between scapular malposition/malorientation and the pitching injuries^5)^. Burkhart et al. proposed the concept of scapular malposition, inferior medial border prominence, coracoid pain and malposition, and dyskinesis of scapula movement (SICK) to explain the scapular asymmetry seen in injured overhead athletes^6)^. However, several studies have reported that overhead athletes have distinctive characteristics in the scapular position and orientation, regardless of shoulder injury^5,7–11^). These results suggest that the SICK scapula may be an adaptive change for efficient pitching performance, rather than a pathological change leading to pitching-related injuries. The scapular dysfunction in pitching motion may be a more reliable predictor of pitching-related shoulder injuries. The concept of hyperangulation^12)^ suggests that the scapula restriction during the cocking phase increases the horizontal abduction of the humerus relative to the glenoid fossa and increases the mechanical stress placed on the shoulder at maximum external rotation (MER) and abduction^12,13)^. It has been clinically recognized that this dynamic malalignment of the scapular–GH complex is associated with pitching-related shoulder injuries^14,15)^. Therefore, the purpose of this study was to individually quantify scapular and GH joint movements during baseball pitching and to compare the biomechanical differences between the groups of baseball pitchers who are prone to shoulder injuries and their counterparts. We hypothesized that injury-prone college baseball pitchers would have less scapular motion and more GH motion when throwing than healthy controls.

## Methods

### Participants

A total of 30 male college baseball pitchers, who pitched in an overhand style, participated in this study. They were members of a college baseball team and had participated in games for 4 years, somewhere between 2012 and 2019. One team athletic trainer provided conditioning and injury prevention programs for these players and maintained daily records of their complaints and conditions. Based on these records, the participants were classified into two groups of 15 participants each as per the injury. The injury-prone group consisted of 14 right-handed and 1 left-handed pitchers, who had sustained repeated nontime-loss shoulder injuries related to pitching within the past 3 months. A nontime-loss injury is defined as an injury that does not result in restriction from participation for at least 24 hrs (no restriction from participation beyond the day of injury), and this definition has been used for injury research in sports^16–18)^. We chose the nontime-loss criterion because the severe pain in the throwing shoulder that causes a time-loss injury may alter the original pitching motion. In this study, all pitchers assigned to this group had experienced two or more episodes of pain from pitching within the 3 months before the measurement. However, the severity of the pain was relatively minor, as they could throw a ball without symptoms the day after therapeutic intervention. We also excluded pitchers who complained of upper extremity pain during the measurement. The control group consisted of 12 right-handed and 3 left-handed pitchers who had no shoulder pain when pitching within the past 6 months. Finally, none of the pitchers in either group had suffered from previous injuries that resulted in surgery or a prolonged absence from the game. Signed informed consent was obtained from each participant before participation in the study. Additionally, the authors obtained informed consent from all participants to publish the motion image in an online open-access publication. The study protocol was approved by the Ethics Committee of Chubu University, Aichi, Japan (Approval Number: 270008).

### Pitching motion analysis

The pitching motion data were collected in an indoor biomechanics laboratory using the procedure previously^19)^. The pitcher’s plate was set up 18 m away from a fixed target (3.0 cm diameter) and placed in the center of a net setup to catch the ball behind a home base. Each participant wore a baseball glove on the nonpitching side and tight-fitting spandex shorts, socks, and shoes with spikes.

**Fig. 1.**
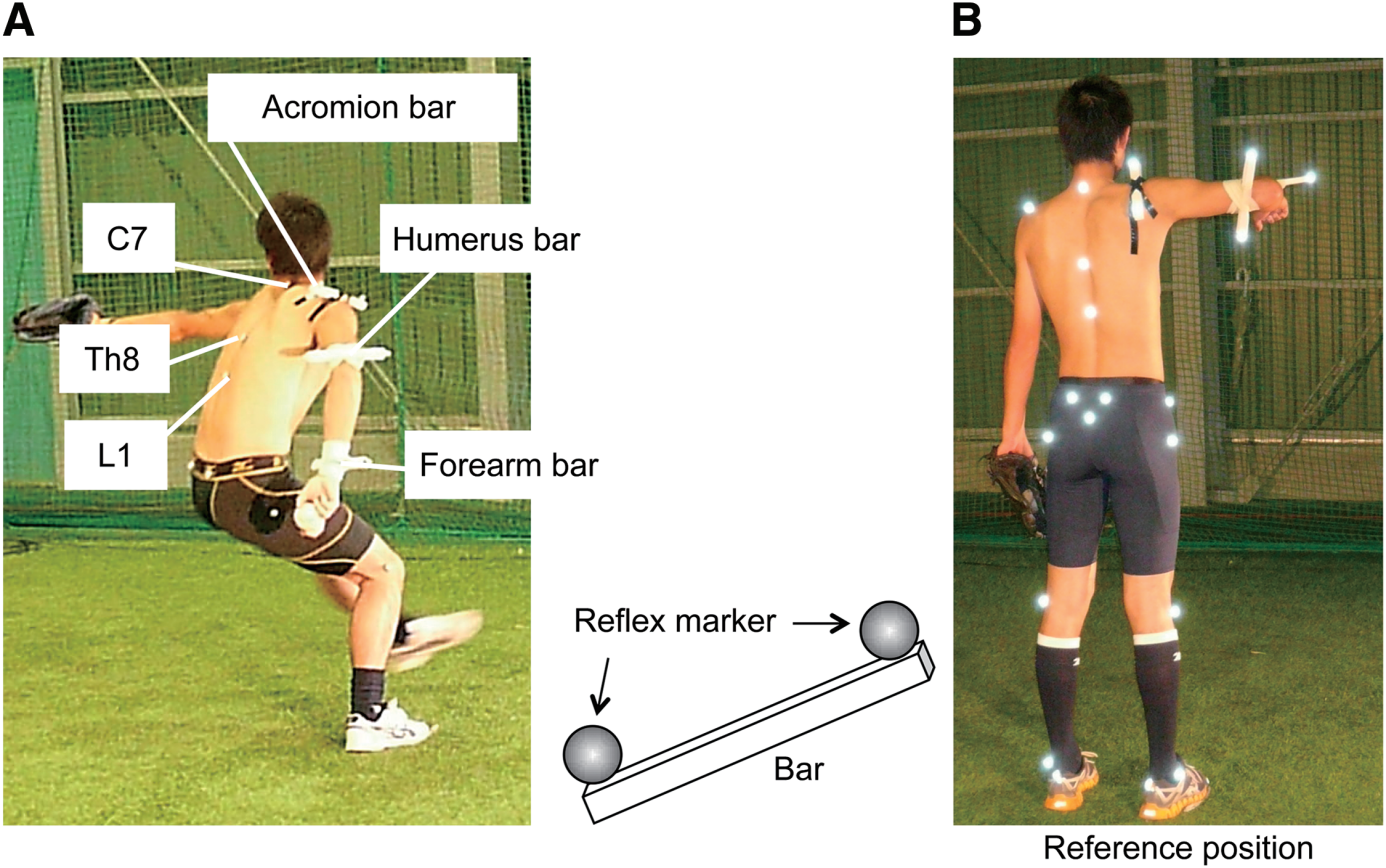
Points for the marker placement and reference position

Figure 1 (left) shows the marker placement in this study. The reflective markers (1.0 cm diameter) were placed at the bony landmarks of the participant as follows: the spinous processes of the seventh cervical (C7), the eighth thoracic (Th8), and the first lumbar (L1) vertebrae, and the manubrium of the sternum. Additionally, lightweight urethane bars (20 cm [length] × 1 cm [width] × 1 cm [height]) with reflective markers attached to both the ends (Fig. 1) were placed on the acromion process, the dorsal side of the distal end of the humerus, and the distal end of the forearm on the pitching side. The acromion bar marker was placed on the flat part of the acromion process so that the two reflective markers affixed on both the ends aligned from front to back. Therefore, the movement of the acromion bar markers indicated the anterior–posterior tilt and external–internal rotation (IR) of the scapula. In addition, two other bar markers were placed on the humerus and forearm to mark the perpendicular direction. To eliminate bar deflection, the bar markers were reinforced with rigid tapes, and they were attached to the center of the bar with a double-sided tape. The affixing site was then crimped with two pieces of high viscoelastic tape, and the two tapes were placed so that they intersected at the center of the bar. Because of the high viscoelasticity of this tape, it stiffened with fast movements, preventing independent movement of the bar relative to the segment in the throwing motion.

(A) The reflective markers were placed at the bony landmarks of the participant as follows: the spinous processes of the seventh cervical (C7), eighth thoracic (Th8), and first lumbar (L1) vertebrae, and manubrium of the sternum. Lightweight urethane bars with reflective markers were affixed on both the edges, on the acromion process, on the dorsal side of the distal end of the humerus, and on the distal end of the throwing forearm. (B) The reference position: the shoulder is 90° flexion and abduction, elbow is 90° flexion, and forearm is in the neutral position.

After a normal warmup, each participant made 10 pitches toward the target with a maximum effort. The pitching motion data were obtained using four high-speed cameras (IEEE 1394b high-speed camera FKN-HC200C; 4 Assist, Tokyo, Japan), and four video images at 200 frames per second were electrically synchronized. The four cameras were placed at the right and left rear, at the dominant hand side, and in the front of the participant. We edited the motion video frame by frame, as shown in the Supplementary S1 movie.

In this study, the most accurate throw to a fixed target among the pitching attempts of each participant was selected for analysis. After transferring the motion data to a personal computer (ESPRIMO WD2/A3; Fujitsu, Tokyo, Japan), the video images were superimposed on a computer display and the markers were automatically tracked using a two- to three-dimensional motion analyzer (Frame-DIAS V; Q’sfix, Tokyo, Japan). Then, the digitized points were obtained using direct linear transformation procedures^20)^. The low-pass filtering was performed at 15 Hz. The computed data were the external rotation (ER) angle of the shoulder complex, the ER angle of the GH joint, the posterior tilting angle of the scapula, the horizontal abduction angle of the shoulder complex, the horizontal abduction of the GH joint, and the ER of the scapula from the stride foot contact (SFC) to the ball release.

**Fig. 2.**
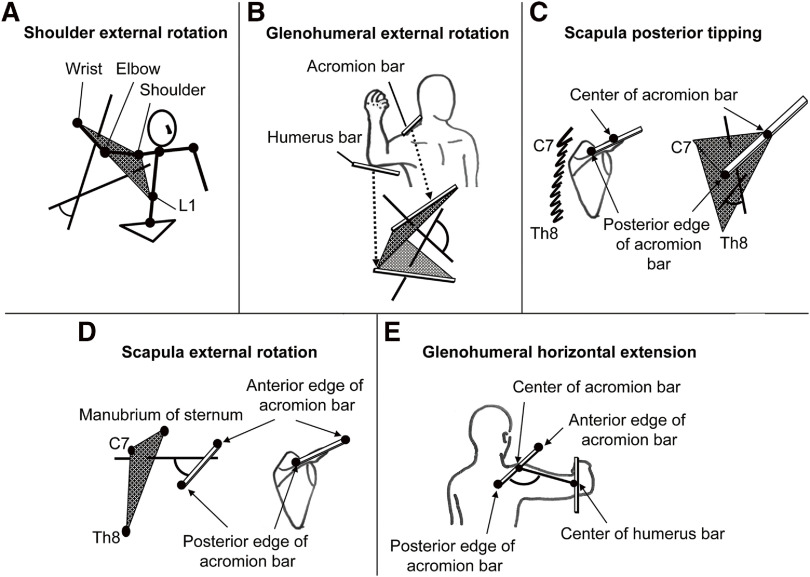
Kinematic model used for the angle calculation

Figure 2 illustrates the kinematic models used in the study^19)^. To calculate the shoulder ER, GH ER, and posterior scapular tilt angles, two corresponding triangles were set up between the markers to define the body segments. For calculating the shoulder ER angle, one triangle was formed between the L1 marker and each midpoint of the acromion bar and humeral bar markers, and another triangle was formed between the midpoints between the forearm marker and the humeral bar and acromion bar markers. Similarly, for calculating the GH ER angle, one triangle was formed between the front and rear markers of the acromion bar and the rear marker of the humeral bar, and another triangle was formed between the front and rear markers of the humeral bar and the rear marker of the acromion bar. Finally, to calculate the posterior tilt angle of the scapula, one triangle was formed between the C7 and Th8 markers and the midpoint of the acromion bar, and another triangle was formed between the midpoint of the acromion bar and the posterior marker and the C7 marker. Once the triangles were formed, the unit vector of the normal direction projected from each corresponding triangle and the inner product of the two unit vectors were calculated. The cosine angle of that inner product was used as each joint angle. For calculating the GH horizontal abduction angle and scapular ER angle, one unit vector of the normal direction projected from the triangle connecting the C7 marker, the Th8 marker, and the median sternal marker was calculated. The scapular ER angle was calculated by the inner product of this unit vector in the normal direction and the acromion bar. The GH horizontal abduction angle was defined as the angle in the horizontal plane between the acromion bar and the line connecting the midpoint of the acromion bar and midpoint of the humeral bar. The cosine angle of the inner product was used as each joint angle. Posterior scapular tilt angle, GH ER, scapular ER, and horizontal scapulohumeral abduction angle were set to 0° when the participant stood upright with shoulder abduction and elbow flexion of 90° (Fig. 1, right). All kinematic data were normalized on a 100% scale from the time of the SFC to the time of ball release to facilitate comparison between participants.

(A) Shoulder ER, (B) GH ER, (C) scapula posterior tipping, (D) scapula ER, and (E) GH horizontal abduction

ER, external rotation; GH, glenohumeral

### Statistical analysis

All calculations were performed using SPSS (version 23.0; IBM, Armork, NY, USA). A two-way repeated measures analysis of variance (ANOVA) was used to examine the main effects of the injury and their interaction on each angle for the injury-prone and control groups at three time points: foot contact, shoulder MER, and ball release. If the interaction was found to be crucial, a post hoc analysis was used, and the Bonferroni test was performed. The alpha level was considered significant when *p* <0.05. Partial eta-squared measure (*η*_*p*_^2^) was used to calculate the effect sizes for ANOVA. The effect size of *η*_*p*_^2^ for the analysis of the covariance test was also calculated^21)^.

The means and standard deviations of the amount of change in each angle during the cocking and acceleration phases were calculated. An unpaired t-test was performed to determine considerable differences between the groups, and values were expressed with a 95% confidence interval (CI). An alpha level was considered significant when *p* <0.05. Cohen’s d effect sizes were used to calculate the effect size for between-group differences. The effect size was interpreted as small (0.20), medium (0.50), and large (0.80)^21)^.

## Results

There was no considerable difference in the mean age and height between the two groups, but there was a considerable difference in body mass between the two groups (Table 1).

**Table 1.** Participant characteristics

	Injury-prone group (n = 15)	Control group (n = 15)	*t*	*p*	*d*
Age, y	20.7 ± 1.4	20.9 ± 1.1	0.311	0.758	0.117
Height, cm	180.1 ± 6.5	177.1 ± 6.6	1.227	0.23	0.464
Mass, kg	78.9 ± 5.4	72.3 ± 6.7	2.968	0.006	1.122

**Fig. 3.**
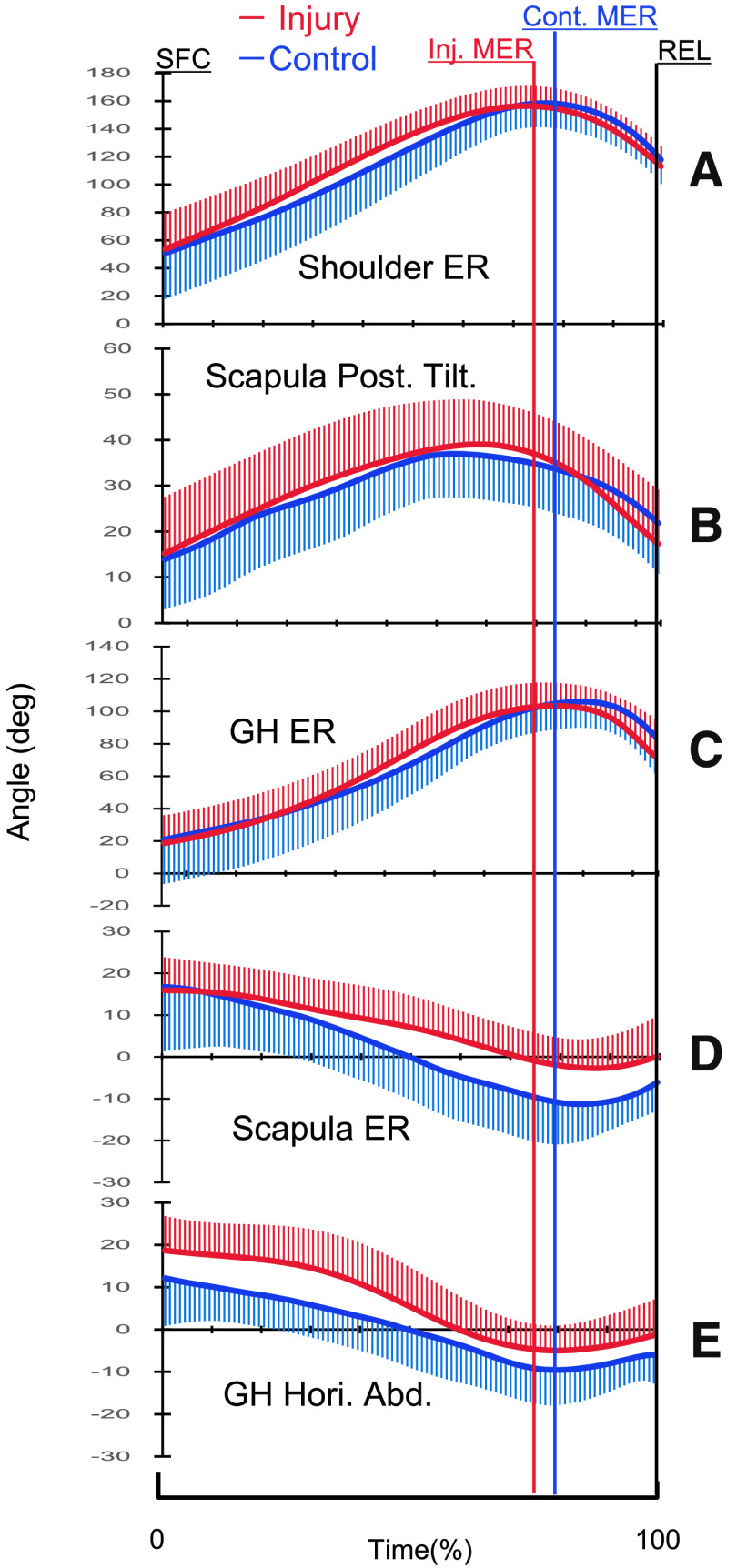
Time curve of the mean (±SD) of joint angle

Figure 3 shows the time curves of each joint angle. There were no considerable differences in shoulder ER angle, scapula posterior tilting, and GH ER angle between the two groups (Tables 2 and 3). The two-way repeated measures ANOVA for scapular ER showed that the main effect of the group was statistically significant (*p* = 0.018, *F* = 6.304, *η*_*p*_^2^ = 0.184), and the interaction effect of injury and phase was significant (*p* = 0.04, *F* = 3.485, *η*_*p*_^2^ = 0.111). Post hoc analysis of the injury × phase interaction effect showed a statistically significant difference in MER (*p* = 0.001, *F* = 13.100, *η*_*p*_^2^ = 0.319) and ball release (*p* = 0.042, *F* = 4.560, *η*_*p*_^2^ = 0.140). In addition, a significant main effect of the group was found in the assessment of the GH horizontal abduction (*p* = 0.006, *F* = 8.769, *η*_*p*_^2^ = 0.238), with no significant interaction between injury and phase (*p* = 0.919, *F* = 0.067, *η*_*p*_^2^ = 0.002).

(A) Shoulder ER angle, (B) scapular Post. Tilt angle, (C) GH ER angle, (D) scapular ER angle, and (E) GH Hori. Abd. in the throwing phase.

SD, standard deviation; ER, external rotation; Post. Tilt, posterior tilting; GH, glenohumeral; GH Hori. Abd., glenohumeral horizontal abduction; MER, maximum external rotation; REL, ball release; SFC, stride foot contact

**Table 2.** Comparison of joint angle for each phase between the injury group and the control group

	Footflat(95% CI)	MER(95% CI)	Release(95% CI)	Interaction			Main effect of group		
				*p*	*F*	Partial *η*^2^	*p*	*F*	Partial *η*^2^
Shoulder ER angle									
Injury-prone group	53.7	157.2	113.2						
	(35.2 to 66.5)	(148.5 to 165.8)	(104.7 to 121.8)	0.622	0.332	0.012	0.736	0.116	0.004
Control group	50.9	160.9	117.8						
	(38.1 to 69.4)	(152.3 to 169.5)	(109.3 to 126.3)						
Scapula posterior tilting angle									
Injury-prone group	15.2	40.5	17.3						
	(9.0 to 21.4)	(35.3 to 45.6)	(11.2 to 23.4)	0.158	1.96	0.066	0.833	3.49	0.001
Control group	14	38.9	21.8						
	(7.8 to 20.1)	(33.6 to 43.8)	(15.8 to 27.9)						
GH ER angle									
Injury-prone group	18.7	102.3	72.1						
	(6.7 to 30.8)	(93.8 to 110.7)	(59.8 to 84.4)	0.408	0.897	0.031	0.332	1.02	0.035
Control group	20.9	102.7	84.8						
	(8.8 to 33.0)	(94.2 to −111.1)	(72.5 to 97.1)						
Scapula ER angle									
Injury-prone group	15.9	−0.1	0.1						
	(9.5 to 22.4)	(−5.0 to 4.8)	(−5.2 to 5.4)	**0.04**	3.49	0.111	**0.018**	6.3	0.184
Control group	16.8	−12.3	−7.7						
	(10.3 to 23.2)	(−17.2 to −7.4)	(−13.0 to −2.4)						
Mean difference	0.8	12.2	7.8						
	(−8.3 to 10.0)	(5.3 to 19.1)	(0.3 to 15.3)						
*p*	0.854	**0.001**	**0.042**						
*F*	0.035	13.100	4.56						
Partial *η*^2^	0.001	0.319	0.14						
GH horizontal abduction									
Injury-prone group	19.0	−4.0	−0.3						
	(14.4 to 23.6)	(−7.7 to 0.2)	(−4.8 to 4.2)	0.919	0.067	0.002	**0.006**	8.77	0.238
Control group	11.7	−10.0	−6.9						
	(7.1 to 16.3)	(−13.7 to −6.3)	(−11.4 to −2.4)						

Angles are shown in degrees.

The significant results are shown in bold fonts.

MER, maximum external rotation; 95% CI, 95% confidence interval; ER, external rotation; GH, glenohumeral

**Table 3.** Comparison of the amount of joint angle change during each phase between the injury group and the control group

	Injury-prone group	Control group	Mean difference (95% CI)	*t*	*p*	*d*
Angle change amount during cocking phase						
Shoulder ER	103.4 ± 27.0	110.0 ± 34.9	6.6 (−16.8 to 29.9)	0.578	0.568	0.219
Scapula posterior tilting	25.2 ± 8.8	24.8 ± 8.5	0.5 (−6.0 to 7.0)	0.164	0.871	0.062
GH ER	83.5 ± 17.3	81.8 ± 31.2	1.7 (−17.1 to 20.6)	0.188	0.852	0.071
Scapula ER	−16.0 ± 6.7	−29.0 ± 21.3	−13.0 (−25.2 to −0.9)	−2.261	**0.037**	0.855
GH horizontal abduction	−23.0 ± 7.5	−21.7 ± 12.2	1.2 (−6.4 to 8.9)	0.334	0.741	0.126
Angle change amount during acceleration phase						
Shoulder ER	−44.0 ± 11.9	−43.0 ± 15.5	0.9 (−9.4 to 11.3)	0.181	0.858	0.068
Scapula posterior tilting	−23.2 ± 7.3	−16.8 ± 9.6	6.3 (−0.0 to 12.7)	2.037	0.051	0.77
GH ER	−30.2 ± 26.3	−17.9 ± 24.8	12.3 (−6.8 to 31.4)	1.320	0.198	0.499
Scapula ER	0.2 ± 10.2	4.5 ± 14.6	4.4 (−5.1 to 13.8)	0.946	0.352	0.358
GH horizontal abduction	3.7 ± 8.2	3.1 ± 6.8	0.6 (−6.2 to 5.1)	0.206	0.838	0.078

Angles are shown in degrees.

The significant result is shown in bold font.

95% CI, 95% confidence interval; ER, external rotation; GH, glenohumeral

In the cocking phase, the amount of angular change in the scapular ER was significantly smaller in the injury-prone group than that in the control group (mean difference, −13.0; 95% CI, −25.2 to −0.9; *p* = 0.037; *d* = 0.855) (Table 3), meaning that the injury-prone group had less IR of the scapula during the cocking and acceleration phases than the control group.

## Discussion

The results of this study showed that the horizontal abduction of the GH joint during the cocking and acceleration phases of the injury-prone group was larger than that of the control group, suggesting that hyperangulation may be a risk factor for pitching-related shoulder injuries^16–18)^. Furthermore, it was shown that the GH joint in the injury-prone group may not have been in a sufficient horizontal adduction position even at the MER. We speculate that in baseball pitchers with such kinematics, various factors such as pitching-related fatigue and shoulder dysfunction may further lead to increased horizontal abduction of the GH joint. Therefore, baseball pitchers who do not have proper scapular positioning during the cocking phases of pitching are considered at risk for injury daily^22,23)^.

The horizontal abduction angle of the GH joint in the release motion was also considerably larger in the injury-prone baseball pitchers. Previous studies on kinematic analysis have reported that the magnitude of anterior force on the shoulder at ball release increases as the shoulder horizontal abduction angle increases^24)^. Therefore, it is possible that the injury-prone group in this study also had repeated pitching-related injuries due to stress from the hyperangulation, as has been presented in previous studies^12)^.

Furthermore, there were differences in the movement of the scapula in the horizontal plane between the injured and healthy control groups. The results showed that there was no considerable difference in the scapular ER angle at the initial contact of the foot between the two groups. However, the injury-prone group had a considerably greater scapular ER angle at MER than the control group. In addition, the amount of IR movement of the scapula during cocking was also considerably smaller in the injury-prone group than that in the control group. In addition, in the injury-prone group, the IR movement of the scapula was considerably smaller during the cocking phase, suggesting that inadequate IR of the scapula during the cocking phase may be associated with the pitching-related shoulder injuries.

Scapula dyskinesis is deemed as a feature of pitching shoulder injuries^6,25)^. It has been suggested that baseball players with the pitching injuries may have the scapula in an internally rotated position and may not be able to adequately externally rotate the scapula during the cocking phase^1,6,25)^, leading to greater horizontal abduction of the GH joint during the subsequent phase. However, the results of our study provide new insights into the relationship between the coordination of the scapula and GH joint and the injury risk. In this study, there was no considerable difference in the scapular ER angle at foot contact, the starting point of the cocking phase, between the two groups. Instead, a difference was found in the subsequent scapular IR movement, suggesting that the inadequate IR of the scapula during the late cocking phases may increase the injury risk rather than the SICK scapular alignment. Based on the flow of the kinetic chain, the serratus anterior muscle plays an important role in the IR movement of the scapular during the cocking and acceleration phases, and therefore, it is important to improve the function of the serratus anterior for preventing the pitching injuries^25)^.

The scapular motion during the pitching motion can also be examined from the perspective of the kinetic chain. In the kinetic chain of pitching, the scapula is responsible for transferring rotational energy from the trunk to the upper extremities^1,25)^, but it can also transfer energy from distal to proximal^26)^. For the scapula to adequately move during the pitching motion and for the kinetic chain to be effective, proper positioning of the upper extremities at foot contact plays an important role. Kreighbaum et al. stated that the inertial resistance of the distal segment “stays in place” while its proximal end is pulled forward by the distal end of the proximal segment^27)^. Conversely, a force of the same magnitude due to inertial resistance is applied to the proximal segment in the opposite direction. Applying this concept to the pitching motion, if the horizontal abduction angle is already large at the time of foot contact, the rearward inertia force due to trunk rotation will be large. Simultaneously, the force applied in the ER direction of the scapula will be large, resulting in a smaller IR of the scapula during cocking in the injury-prone group than that in the control group. As a result, the kinetic chain between the scapula and GH joint, as indicated by the “lagging-back phenomenon” that is a kinetic chain phenomenon in which the distal part of the body (humerus) lags behind the proximal segment (scapula) toward acceleration^27)^, may not have efficiently worked. Because the distal segment lags larger behind the injury-prone pitchers, the stress on the shoulder joint may increase compared to healthy counterparts. From the perspective of the kinetic chain of the throwing motion, it is necessary to focus on the cocking motion from foot contact when pitching to prevent injuries.

On the other hand, in this study, there were no considerable group differences in posterior scapular tilt and GH ER motion in all phases. It is well known that most of the pitching injuries occur in the phase close to the MER. Fleisig et al. reported that an IR torque of 67 N·m and an anterior shear force of 310 N are applied to the shoulder just before the MER in the pitching motion^28)^. Sabick et al. also reported that the peak humeral axial torque reached a mean value of 92 ± 16 N·m near the point of MER^29)^. These previous findings indicate that ER during the pitching motion places a great deal of stress on the GH joint and can be a major cause of pitching injury. However, in this study, there was no difference in the magnitude of the MER angle. Although not analyzed statistically at this time, there was a slight difference in the timing of this MER between the two groups, as shown in Figure 3. Whiteley stated that the stress on the GH joint varies greatly depending on the “timing of horizontal abduction and ER,” and when the GH ER is forced into the horizontal abduction position, the risk of the pitching-related shoulder injuries may be increased^30)^. The relative positions of the horizontal abduction and ER of the GH during pitching need to be further investigated to determine how they affect stress on the shoulder.

A major limitation of this study is the difficulty in eliminating measurement errors in the movement of the scapula during the pitching motion. The first is that the movement of the scapula under the skin was indirectly measured rather than directly. To minimize the measurement error due to skin artifacts, the acromion, which has little skin movement in previous studies^31,32)^, was selected as the application site in this study. In addition, the scapular external/IR angles associated with horizontal extension and flexion movements calculated with our method were visually compared with the scapular angles measured with electromagnetic sensors in the study by Bourne et al. to indirectly confirm the validity of our method^33)^. We also confirmed that the shoulder joint ER and the horizontal abduction angle of the shoulder during pitching obtained by the current method were consistent with those obtained by a three-dimensional motion analysis system equipped with an electromagnetic tracking device^34,35)^. The pattern of angle change in the scapula ER angle from the SFC to ball release in the pitching motion was also found to be similar to that in previous studies^34,35)^. Therefore, although the scapular movement measurement method in this study may be less accurate than the direct method, it does not appear to have a considerable effect that would change the considerably differences found between the control and injury-prone groups. Moreover, as shown in the video material, the bar markers allowed the indirect visualization of the scapula movement during the pitching motion and reinforced the validity of the angle-change curves.

This study demonstrated that there were differences between the injury-prone and healthy counterparts in the movement of the scapula and GH joint during the pitching motion. Three-dimensional analysis revealed that the injury-prone group had a considerably greater horizontal abduction angle than the control group. Furthermore, there was a difference in the movement of the scapula on the horizontal plane between the groups; the injury-prone group had a greater ER angle of the scapula than the control group in MER and ball release, but there was no difference in foot contact between the groups. Moreover, the amount of decrease in the scapular ER angle during the cocking phase was considerably smaller in the injury-prone group than that in the control group, suggesting that injured baseball pitchers do not have sufficient control over scapular IR during the late cocking phase. To establish effective preventive measures against the pitching-related shoulder injuries, further research is needed to determine whether the biomechanical characteristics of the scapula and GH joint in the injury-prone pitchers are the result of dysfunction of the scapula or a problem in the kinetic chain during pitching.

Finally, future issues are presented. In this result, some differences in joint movement were observed between the injury-prone group and the control group. Therefore, it is necessary to clarify the factors that cause this difference. In the field of baseball, regardless of the presence or absence of disability, there are many differences in scapula motion between the pitching side and the non-throwing side. While this difference can be evaluated as scapula dyskinesis^6)^ that causes throwing injuries, but it is also possible that it is an adaptation^5)^ to pitching motion. In addition, the joint functions of baseball players are changing day by day. Therefore, the reality is that a demarcation line between functional decline and adaptation is not clear. All subjects in this study also had no pain in the shoulder when shooting the pitching motion, but most of the players had a left–right difference in the scapular movement, and the differences were very diverse. Therefore, it is necessary to analyze not only scapular function but also other aspects of factors that cause differences in scapular movement during pitching. The factors may ultimately have to be evaluated by individual players.

## Conclusions

This study demonstrated the biomechanical differences in the scapular and GH movements during pitching between 15 injury-prone pitchers and 15 healthy college baseball pitchers. The horizontal abduction angles of the GH joint during cocking and acceleration phases were considerably greater in the injury-prone pitchers than those in healthy controls. Moreover, in the cocking phase, the amount of angular change in the scapular ER was significantly smaller in the injury-prone group than that in the control group. These results suggest that the injury-prone pitchers have less IR of the scapula and a more horizontal abduction of the GH joint during the cocking and acceleration phases. Therefore, sports medicine practitioners may need to pay considerable attention to the coordination of scapular and GH horizontal movements during pitching for prevention of shoulder injuries.

## Data Availability

The datasets generated and/or analyzed during this study are available from the corresponding author on reasonable request.

## Acknowledgments

The authors would like to thank Enago (www.enago.jp) for the English language review. This research did not receive any specific grant from funding agencies in the public, commercial, or not-for-profit sectors.

## Funding

This research did not receive any specific grant from funding agencies in the public, commercial, or not-for-profit sectors.

## Conflict of Interest

The authors declare no competing interests.

## Supplementary Material

Supplementary S1 movie. Typical example of motion video for each frame.
